# On some new species of Ancorabolidae Sars, 1909 from the Gulf of California: the genera *Ceratonotus* Sars, 1909, and *Dendropsyllus* Conroy-Dalton, 2003 (Crustacea, Copepoda, Harpacticoida)

**DOI:** 10.3897/zookeys.657.10725

**Published:** 2017-02-17

**Authors:** Samuel Gómez, Karen Díaz

**Affiliations:** 1 Instituto de Ciencias del Mar y Limnología, Unidad Académica Mazatlán, Universidad Nacional Autónoma de México; Joel Montes Camarena s/n, Fracc. Playa Sur, Mazatlán, 82040,Sinaloa, México; 2 Posgrado en Ciencias del Mar y Limnología, Unidad Académica Mazatlán, Universidad Nacional Autónoma de México; Joel Montes Camarena s/n, Fracc. Playa Sur, Mazatlán, 82040, Sinaloa, México

**Keywords:** Deep sea, Gulf of California, Harpacticoida, taxonomy

## Abstract

Two new species of two genera of the family Ancorabolidae, *Ceratonotus elongatus* **sp. n.** and *Dendropsyllus californiensis* **sp. n.**, found at 1642 m and 1759 m depth, respectively, in the Southern Trough of Guaymas Basin, are described. *Ceratonotus elongatus* **sp. n.** was attributed to that genus by a series of character states of which the lack of dendroid dorsal processes on the P6-bearing somite and the presence of such processes on the first abdominal somite were definitive. This species was observed to be very close to *Ceratonotus thistlei* Conroy-Dalton, 2003 from the San Diego Trough, and can be separated by a number of traits of which the elongated sensilla-bearing dorsal tubercles on the second abdominal somite in the new species was definitive. *Dendropsyllus californiensis* **sp. n.** has been placed within that genus given the presence of four geniculate setae on P1EXP2 and one seta on P1ENP2, one inner seta on P3EXP3, and lack of inner armature on P4EXP3. *Dendropsyllus californiensis* **sp. n.** appears to be more closely related to *Dendropsyllus thomasi* Conroy-Dalton, 2003 and *Dendropsyllus magellanicus* (George & Schminke, 1998) on account of the spinulose nature of the basis of the maxilliped, the two-segmented P4ENP, and the fused condition of the P5 baseoendopod and exopod, and seems to be even more closely related to *Dendropsyllus thomasi* by the degree of development of the lateroventral processes of the cephalothorax. *Dendropsyllus californiensis* **sp. n.** can be separated from its congeners by the relative length of the first antennulary segment, relative length of the caudal rami, and by the armature formula of P3ENP2.

## Introduction

**Figure 1.**
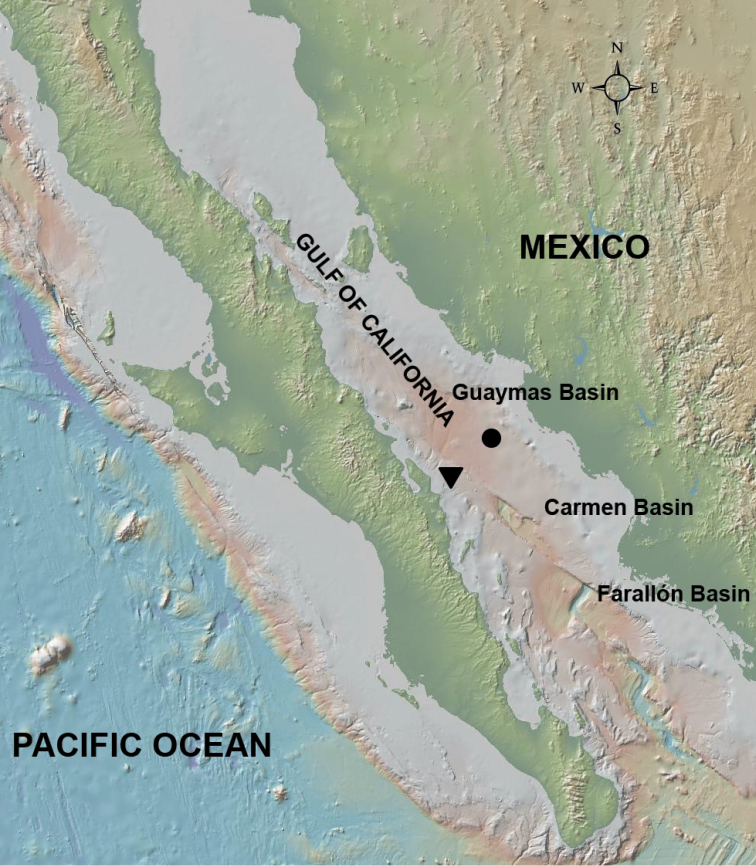
Sampling sites and type localities of *Ceratonotus elongatus* sp. n. (circle) and *Dendropsyllus californiensis* sp. n. (inverted triangle). Figure prepared with GeoMapApp (http://www.geomapapp.org/) and the Global Multi-Resolution Topography (GMRT) default basemap (Ryan et al. 2009).

The macrofauna diversity of the Gulf of California is fairly well known. The Gulf is home to more than 4,916 named species of macroinvertebrates, comprising approximately 70% of the invertebrate fauna of the Gulf of California. In contrast, the open sea and the deep sea below the continental shelf are regions more poorly known (Brusca and Hendrickx 2012). For example, a quick search in The Gulf of California Invertebrate Database (Brusca and Hendrickx 2008) yielded only around 508 benthic macroinvertebrate species reported below 200 m depth. The biodiversity of meiofauna of the Gulf of California is even less known, and is based on few studies available, mostly from sublittoral situations (e.g. Álvarez-Castillo et al. 2015 for kinorhynchs, Holovachov et al. 2008, 2009, Mundo-Ocampo et al. 2007, Pereira et al. 2010 for nematodes, and see Gómez and Morales-Serna 2014 for harpacticoid copepods), while only a handful of described meiofaunal species are known from the deep Gulf of California below 200 m depth (e.g. Álvarez-Castillo et al. 2015 for kinorhynchs, and both Gómez and Conroy-Dalton 2002 and Gómez and Morales-Serna 2014 for harpacticoids). The low number of studies on the diversity of deep-sea meiofauna of the Gulf of California is due mainly to a difficult taxonomy, special sample processing techniques required for the different taxa, and above all to a limited expertise and lack of experts (Gómez and Morales-Serna 2014, Álvarez-Castillo et al. 2015, Pereira et al. 2010). A series of intensive oceanographic cruises, Talud IV-XVI, have been carried out in the deep-sea of the Gulf of California since the late 90’s. These samplings include, among other components, the meiofaunal communities, of which harpacticoid copepods are the only meiofaunal group studied so far with one described species (*Ancorabolus hendrickxi* Gómez & Conroy-Dalton, 2002), from a depth of 1985 m off Sinaloa state (Gómez and Conroy-Dalton 2002). Described herein are two more species of two genera of the family Ancorabolidae, *Ceratonotus elongatus* sp. n. and *Dendropsyllus californiensis* sp. n. found at 1642 m and 1759 m depth in the Southern Trough of Guaymas Basin (see Fig. 1), with closely related species, *Ceratonotus thistlei* Conroy-Dalton, 2003 and *Dendropsyllus thomasi* Conroy-Dalton, 2003, respectively, from the San Diego Trough.

## Material and methods

Sediment samples for meiofaunal analyses were taken in February 2007 during the Talud X oceanographic cruise in the Southern Trough of Guaymas Basin, on board the research vessel “El Puma” of the Universidad Nacional Autónoma de México (UNAM). The sediment samples were collected using a box corer, and triplicate sub-samples were taken with 69 cm^2^ cores of 20 cm in length. The upper 3 cm layer of sediment was recovered and preserved in 70% alcohol, sieved through 500 and 38 µm sieves to separate macro- and meiofauna, and stained with Rose Bengal. Meiofauna was sorted and quantified at a magnification of 40× using an Olympus SZX12 stereomicroscope equipped with DF PLAPO 1× objective and WHS10X eyepieces. The specimens of the species presented herein were partly dissected as indicated in “Material examined” for each species. Illustrations and figures were made from whole individuals and its dissected parts using a Leica DMLB microscope equipped with L PLAN 10× eyepieces, N PLAN 100× oil immersion objective, and drawing tube. The dissected parts were mounted on separate slides using lactophenol as mounting medium. Terminology of Huys and Boxshall (1991), Conroy-Dalton (2003), and George (2006b) were adopted for descriptive morphology. Abbreviations used in the text:

P1-P6
 first to sixth legs;


EXP
 exopod;


ENP
 endopod;


EXP(ENP)
 1(2,3) first (second, third) exopodal (endopodal) segment;


ae
 aesthetasc;


**mya** million years ago.

The type material was deposited in the Copepoda collection of the Instituto de Ciencias del Mar y Limnología, Unidad Académica Mazatlán (**ICML-EMUCOP**).

The map showing the sampling locations where the new species were found were prepared with GeoMapApp (http://www.geomapapp.org/) and the Global Multi-Resolution Topography (GMRT) default basemap (Ryan et al. 2009).

## Results

### Taxonomy

#### Family Ancorabolidae Sars, 1909

##### Subfamily Ancorabolinae Sars, 1909

###### Genus *Ceratonotus* Sars, 1909

####### Ceratonotus elongatus sp. n.

Taxon classificationAnimaliaHarpacticoidaAncorabolidae

http://zoobank.org/1A041782-E9FC-4455-9D57-82A8A2A07DE1

######## Material examined.

One female holotype as follows: body partially dissected (leaving cephalothorax with right first antennulary segment and antenna, first thoracopod to fifth urosomite, and right P5 intact) and preserved in alcohol (ICML-EMUCOP-100207-01), left antennule and antenna, pair of mandibles, maxillules, maxillae and maxillipeds, P1-P4 and left P5, and anal somite with caudal rami dissected and mounted on four slides (ICML-EMUCOP-100207-04).

######## Type locality.

Southern Trough of Guaymas Basin, Gulf of California, México (27°01'N, 110°53'04"W), 1642 m depth (see Fig. 1); coll. S. Gómez.

######## Diagnosis

(based on the female only). Ancorabolidae. Cephalothorax without anterior horn-like processes, with bilateral constriction in anterior half. First half of genital double-somite without, posterior half with well-developed dendroid processes. With dorsal elongate tubercles and paired tube-pores on fourth urosomite. Caudal rami divergent, approximately 11 times as long as wide, with seven setae of which seta IV fused at base of seta V. Antennule three-segmented. Antenna with allobasis bearing one abexopodal seta; without exopod; endopod with nine setae/spines. Palp of mandible one-segmented, with five setae. Maxillule with two surface setae and seven spines on praecoxal arthrite; coxal endite with two elements; basis with six setae; exopod represented by two, endopod by three elements. Maxilla with two syncoxal endites, each with three setae; endopod represented by two setae. Maxilliped with one seta on syncoxa; endopodal claw with one accessory seta. Exopod of P1 two-segmented, of P3-P4 three-segmented. Endopod of P1-P4 two segmented; endopod of P1 as long as exopod, second endopodal segment approximately 1.7 times as long as first endopodal segment; first endopodal segment of P2 and P3 reduced, smaller than second endopodal segment; endopod of P4 much smaller than in P2 and P3, first endopodal segment twice as long as second. P5 with exopod and endopodal lobe distinct; endopodal lobe a tiny pedestal with one seta and one tube-pore; exopod elongate, slender, with three elements.

######## Description of female.

**Figure 2.**
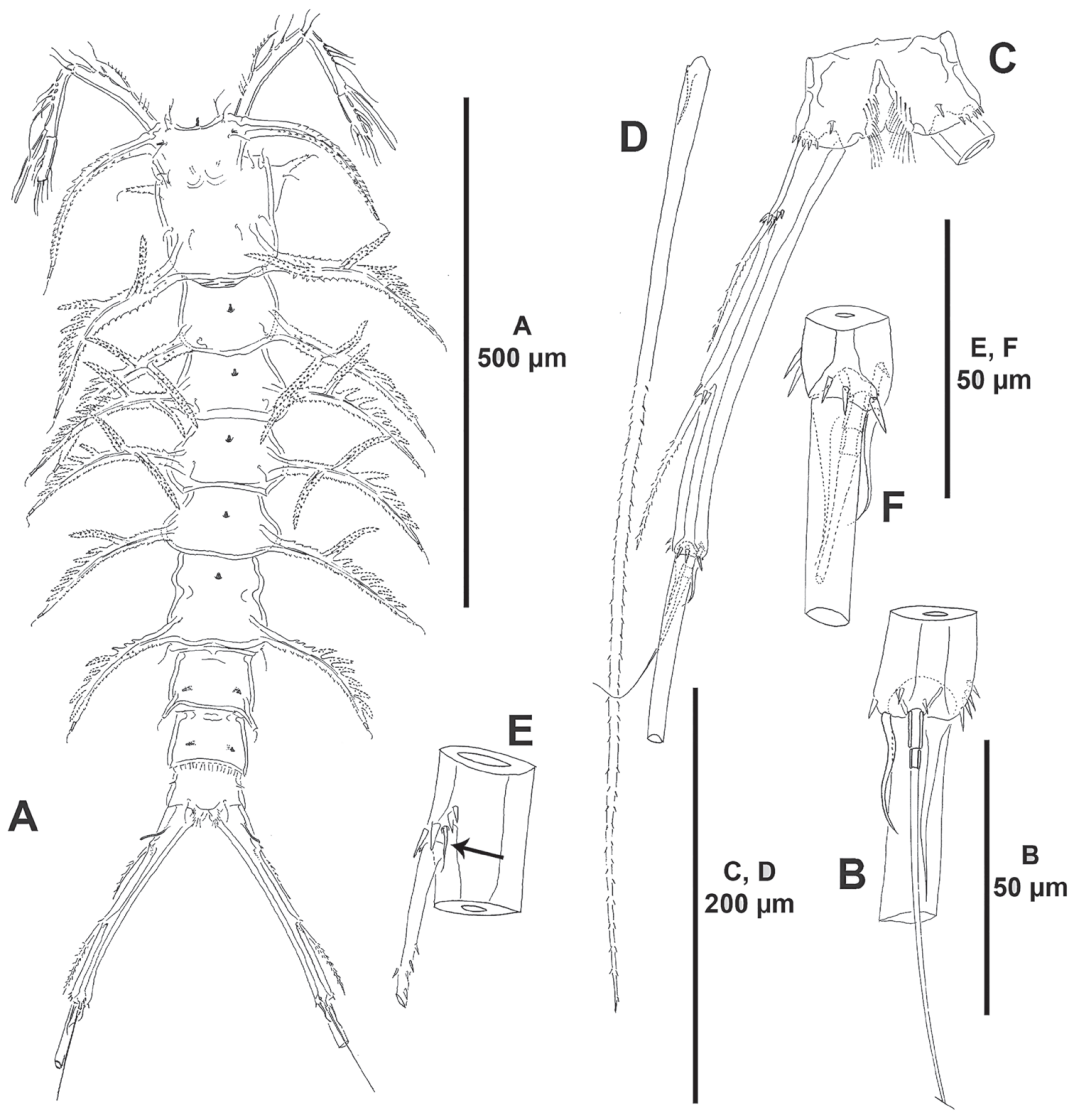
*Ceratonotus elongatus* sp. n., female holotype. **A** habitus, dorsal **B** distal part of caudal ramus, dorsal **C** anal somite and right caudal ramus, ventral **D** caudal setae IV and V **E** caudal setae I and II, indicating position of reduced seta I **F** distal part of caudal ramus, ventral.

**Figure 3.**
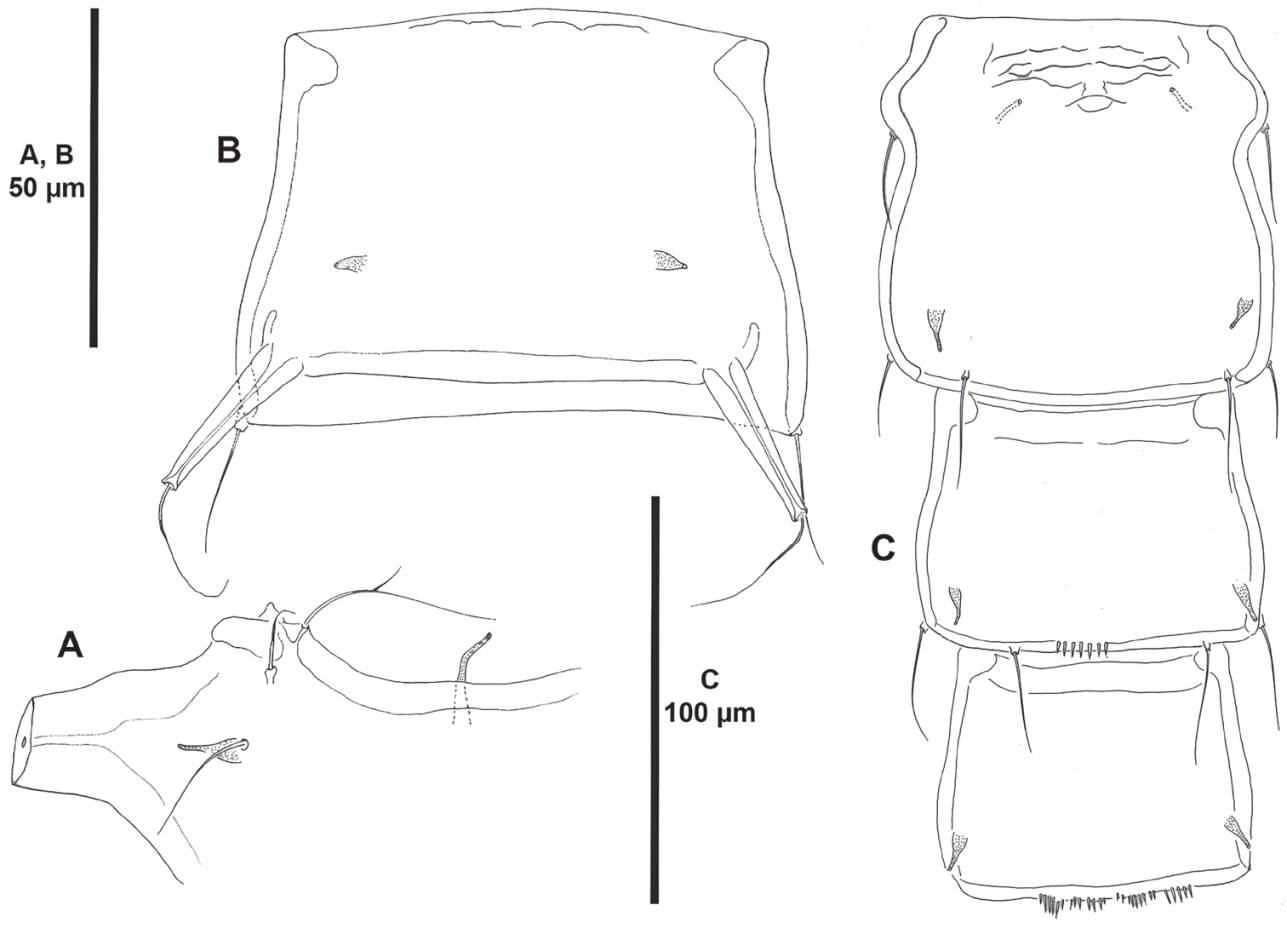
*Ceratonotus elongatus* sp. n., female holotype. **A** anterior corner of cephalothorax **B** fourth urosomite (second abdominal somite), dorsal **C** genital double-somite, and fourth and fifth urosomites (second and third abdominal somites), ventral.

Total body length, 920 µm measured from anterior outer corner of cephalothorax to posterior margin of caudal rami; length of caudal rami, 222.5 µm (ca. 24% total body length). Body (Fig. 2A) cylindrical, rather slender, tapering slightly posteriorly, without clear demarcation between prosome and urosome; integument moderately chitinised; general pattern of dendroid processes as for the genus; dendroid processes well developed. Cephalothorax without anterior horn-like processes, with bilateral constriction in anterior half; with large tube-pore medially along anterior margin; anterior corners of cephalothorax with two anterior sensilla associated with a tube-pore (Fig. 3A); dorsal dendroid processes well developed (Fig. 2A); lateroventral processes situated rather anteriorly. Rostrum fused to cephalothorax, absorbed into anteroventral surface of cephalothorax, with paired sensilla and well-developed midventral tube-pore (Figs 2A, 3A).

P2-P4-bearing somites with medial tube-pore and two posterior sensilla; dorsal dendroid processes well developed. P5-bearing somite with dorsal tube-pore, without posterior sensilla; with well-developed dorsal processes, nearly as long as in preceding somites and without backwardly directed excrescent.

Original segmentation of genital double-somite indicated by bilateral constriction; first half of genital double-somite with dorsal tube-pore, without dendroid processes, without spinular ornamentation ventrally, genital field as shown (Fig. 3C); posterior half without dorsal tube-pore, with well-developed dendroid process, though smaller than those of P5 bearing-somite (Fig. 2A), ventrally without spinular ornamentation but with paired tube-pores (Fig. 3C). Fourth urosomite (second abdominal somite) with elongate tubercles and paired tube-pores dorsally (Figs 2A, 3B), ventrally with medial short spinular row close to posterior margin and with paired tube-pores (Fig. 3C). Fifth urosomite (third abdominal somite) with paired tube-pores and with fine spinules along posterior margin dorsally (Fig. 2A), with four medial sets of spinules and paired tube-pores ventrally (Fig. 3C). Anal somite cleft medially (Fig. 2A, C); with tube-pore and small spinules ventrally as shown (Fig. 2C); rounded anal operculum smooth (Fig. 2A).

Caudal rami elongate, divergent, cylindrical, approximately 11 times as long as wide (Fig. 2A, C); with some spinules at base of setae I, II and III, and close to posterior margin ventrally; with conspicuous tube-pore proximally (Fig. 2A); with seven setae; seta I minute, ventral to seta II (Fig. 2A, C,E), both situated on proximal fifth of ramus; seta II bipinnate; seta III inserted on proximal margin of distal third, as long as seta II, bipinnate (Fig. 2A, C); seta IV fused at base of seta V (Fig. 2B, D, F), the latter longest, ornamented as shown (Fig. 2D); seta VI shorter than seta IV, inserted on distal inner corner of ramus (Fig. 2B, C, F); dorsal seta VII triarticulate, arising from small pedestal close to posterior margin (Fig. 2B).

**Figure 4.**
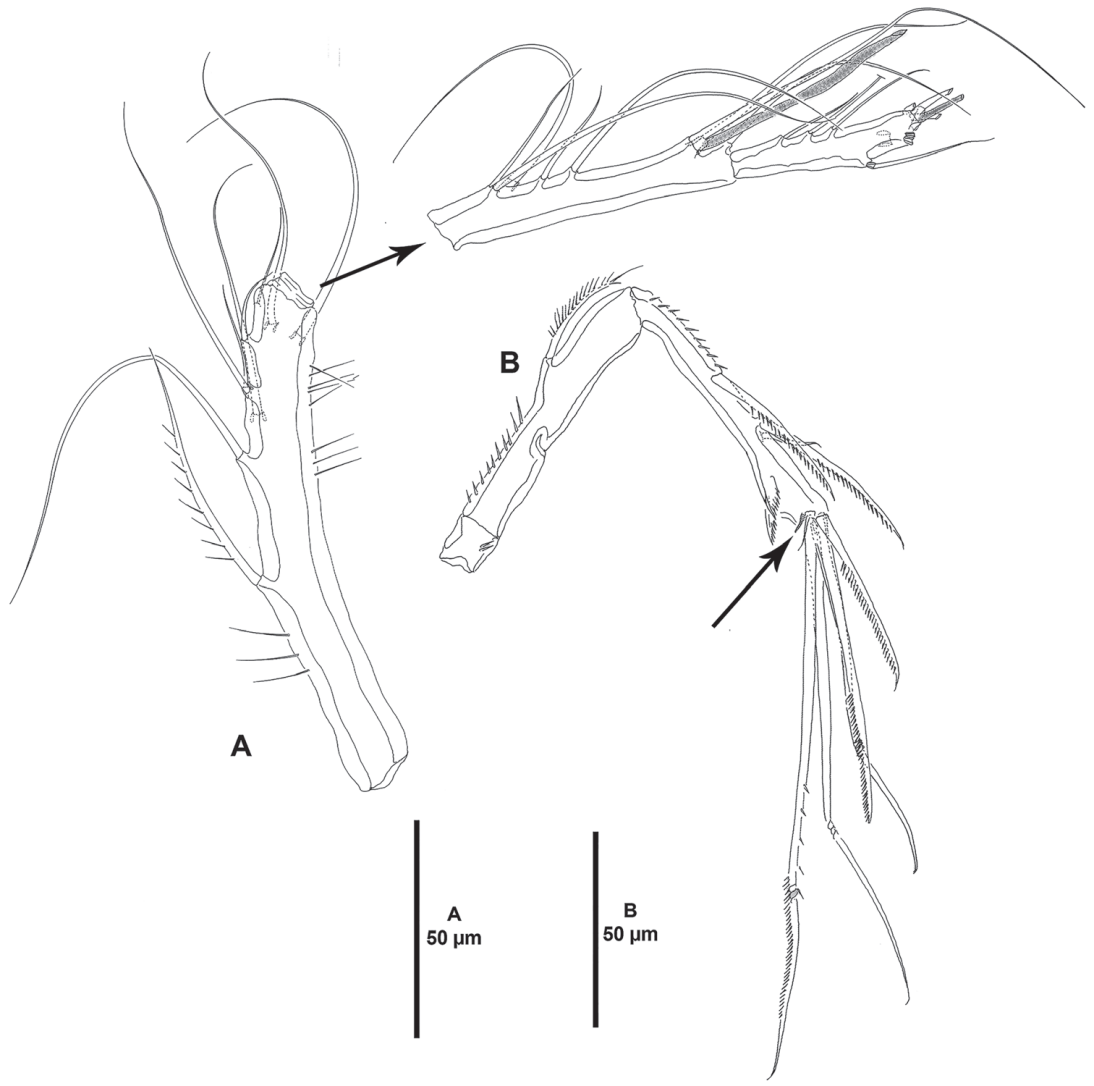
*Ceratonotus elongatus* sp. n., female holotype. **A** antennule **B** antenna.

Antennule (Fig. 4A) three-segmented, segments elongate and slender. Armature formula as follows: 1-[9], 2-[7+(1+ae)], 3-[9+acrothek].

Antenna (Fig. 4B). Coxa represented by sclerite; with allobasis and one-segmented endopod. Allobasis with membranous insert indicating original division between basis and first endopodal segment; with small spinules along inner margin of proximal half; with well-developed pinnate abexopodal seta in endopodal half. Exopod absent. Endopod with small spinules along inner margin of proximal half; with two inner lateral spines and one slender seta; with two outer subdistal frills; apically with two pinnate spines, two geniculate single setae, and one geniculate element fused to tiny seta basally; with additional distal tube-pore (arrowed in Fig. 4B).

**Figure 5.**
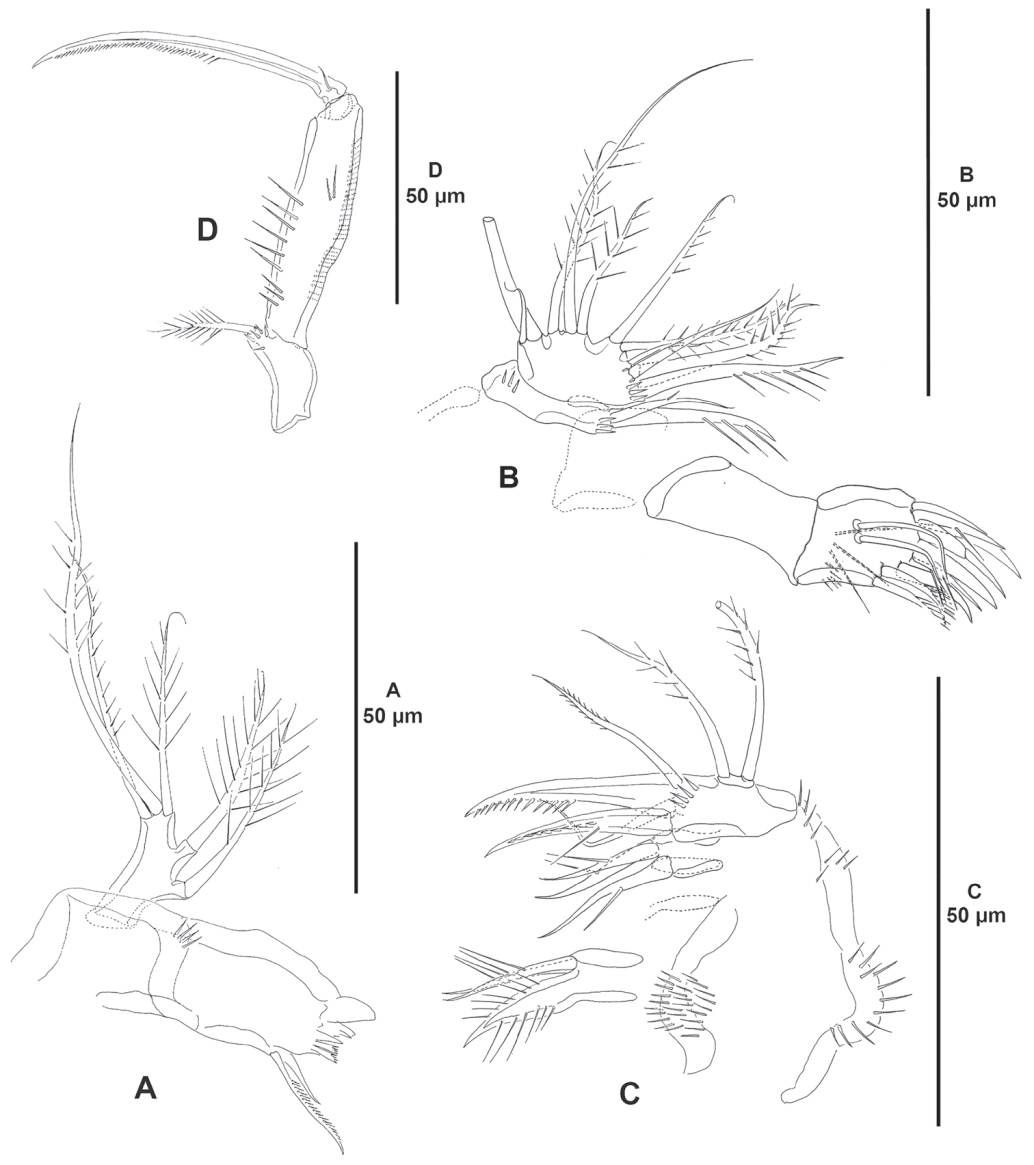
*Ceratonotus elongatus* sp. n., female holotype. **A** mandible **B** maxillule **C** maxilla, showing insertion of proximal endite **D** maxilliped.

Mandible (Fig. 5A) with robust coxa. Gnathobase with distal teeth as shown, with one lateral pinnate seta accompanied by spine-like element. Palp one-segmented, well developed, with two inner (basal) setae, and three apical (endopodal) setae.

Maxillule (Fig. 5B). Praecoxal arthrite with two surface setae and some posterior spinules (some of them very long), distally with seven spines (two of them spinulose) and two pinnate setae. Coxal endite with some spinules distally, with one strong and spinulose element and one slender seta ornamented with very few spinules. Proximal endite of basis with four, distal endite with two setae. Exopod and endopod incorporated into basis, the former represented by one small and one strong seta, the latter represented by three pinnate elements.

Maxilla (Fig. 5C). Syncoxa with spinulose patches as shown, with two endites; proximal endite with one strong spinulose element fused to endite, and two spinulose setae, distal endite with three spinulose elements. Allobasis drawn out into strong claw; accessory armature consisting of one spinulose strong spine, and one bare and one pinnate seta. Endopod represented by two setae.

Maxilliped (Fig. 5D) subchelate, slender. Syncoxa with one pinnate seta. Basis with spinules as figured. Endopod drawn into long, curved claw finely pinnate with one accessory small seta.

**Figure 6.**
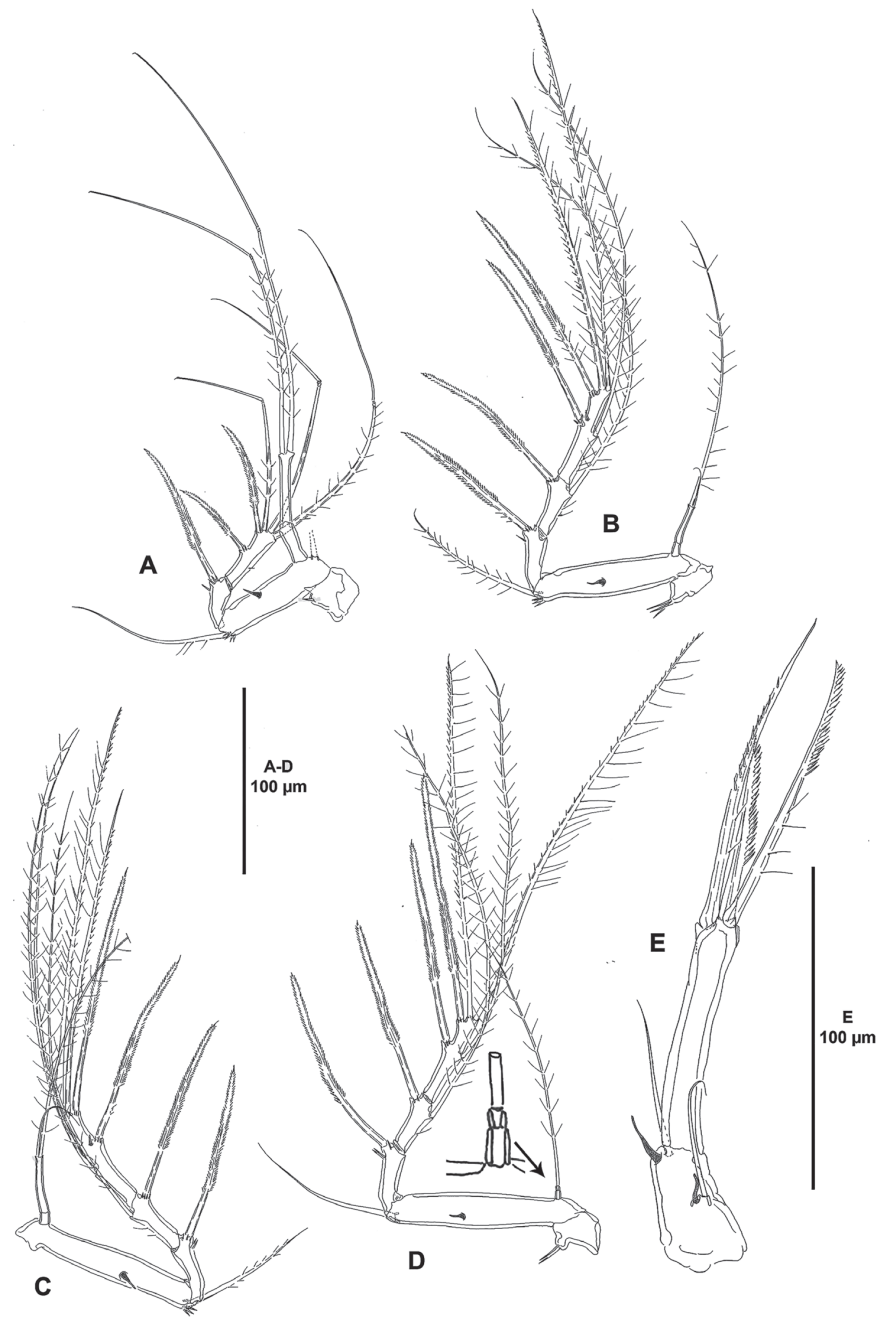
*Ceratonotus elongatus* sp. n., female holotype. **A** P1, anterior **B** P2, anterior **C** P3, anterior **D** P4, anterior, showing insert of endopod **E** P5, lateral.

P1 (Fig. 6A). Coxa with one outer spinule. Basis transversely elongate, with anterior tube-pore, with some spinules at base of outer seta, the latter well-developed, inner seta lost during dissection. Exopod two-segmented; first segment with pinnate spine longer than entire ramus; second segment with two pinnate outer spines and three geniculate apical setae. Endopod two-segmented, nearly as long as exopod; first segment unarmed; second segment 1.7 times as long as first one, with two apical setae.

P2-P4 (Fig. 6B–D) with trapezoid coxa ornamented with spinules on lobate outer process. Basis transversely elongate, with anterior tube-pore midway length of basis, with some spinules at base of outer seta. Exopod three-segmented; first segment with outer bipinnate elongate spine; second segment with outer bipinnate elongate spine and inner seta; third segment with two outer elongate spines, two apical elements and one (P2 and P4) or two (P3) inner setae, with (P2 and P3) or without (P4) tube-pore. Endopod two-segmented; P2ENP1 and P3ENP1 reduced, smaller than ENP2, the latter with two setae, inner one smaller; P4ENP much smaller than in preceding legs, ENP1 twice as long as ENP2, the latter with one seta.

Armature formula as follows:

**Table:** 

	EXP	ENP
P1	0.023	0.020
P2	0.1.122	0.020
P3	0.1.222	0.020
P4	0.1.122	0.010

P5 (Fig. 6E) without baseoendopodal setophore; outer basal seta bare, accompanied by tube-pore. Endopodal lobe represented by tiny pedestal armed with one seta and accompanied by tube-pore. Exopod distinct, long, slender, with three elements as figured.

######## Description of male.

Unknown.

######## Etymology.

The specific epithet, *elongatus*, makes reference to the elongate dorsal tubercles on the second abdominal somite.

###### Genus *Dendropsyllus* Conroy-Dalton, 2003

####### Dendropsyllus californiensis sp. n.

Taxon classificationAnimaliaHarpacticoidaAncorabolidae

http://zoobank.org/4730BB01-67A4-4AF9-9942-27E5FA1B3580

######## Material examined.

One female holotype as follows: body partially dissected (leaving cephalothorax, left antennule and antenna, left P1-P5, abdomen, anal somite and caudal rami intact) and preserved in alcohol (ICML-EMUCOP-100207-02), pair of mandibles, maxillules, maxillae and maxillipeds, and right P1-P5 dissected and mounted on four slides (ICML-EMUCOP-100207-03).

######## Type locality.

Southern Trough of Guaymas Basin, Gulf of California, México (26°41'06"N, 111°12'W), 1759 m depth (see Fig. 1); coll. S. Gómez.

######## Diagnosis

(based on the female only). Ancorabolidae. Cephalothorax with bilateral anterior constriction; with two sensilla and one tube-pore on distal corners; with paired dorsal processes anteriorly, lateroventrally, and posteriorly. Rostrum fused to cephalothorax. P2-P5-bearing somites with paired dorsal dendroid processes. Second and third urosomites fused ventrally, distinct dorsally, without dendroid processes. Caudal rami divergent, around 7.5 times as long as wide; with seven setae. Antennule three-segmented. Antenna with allobasis bearing a reduced abexopodal seta; without exopod; free endopodal segment with eight setae/spines. Mandible with one-segmented palp bearing five setae. Maxillule with two surface setae and five spines on praecoxal arthrite; coxal endite with two setae; basis with six setae; exopod represented by two, endopod by three elements. Maxilla with two syncoxal endites bearing three setae each; allobasis drawn out into strong claw, accompanied by five elements; endopod one-segmented, with two setae. Maxilliped with one seta on syncoxa; endopodal claw with one accessory seta. Exopod of P1 two-segmented, of P2-P4 three-segmented. First endopodal segment of P1 small, second segment elongate, close to 4.3 times as long as first segment, and 7.6 times as long as wide. P2 without endopod. First endopodal segment of P3 and P4 very small, second segment around 8.6 and 4.4 times as long as first segment, and 8.6 and 4 times as long as wide, respectively. P5 with baseoendopod and exopod fused; endopodal lobe a small pedestal with one naked seta and one tube-pore; exopod slender, 7.7 times as long as wide, with long subdistal tube-pore and three elements.

######## Description of female.

**Figure 7.**
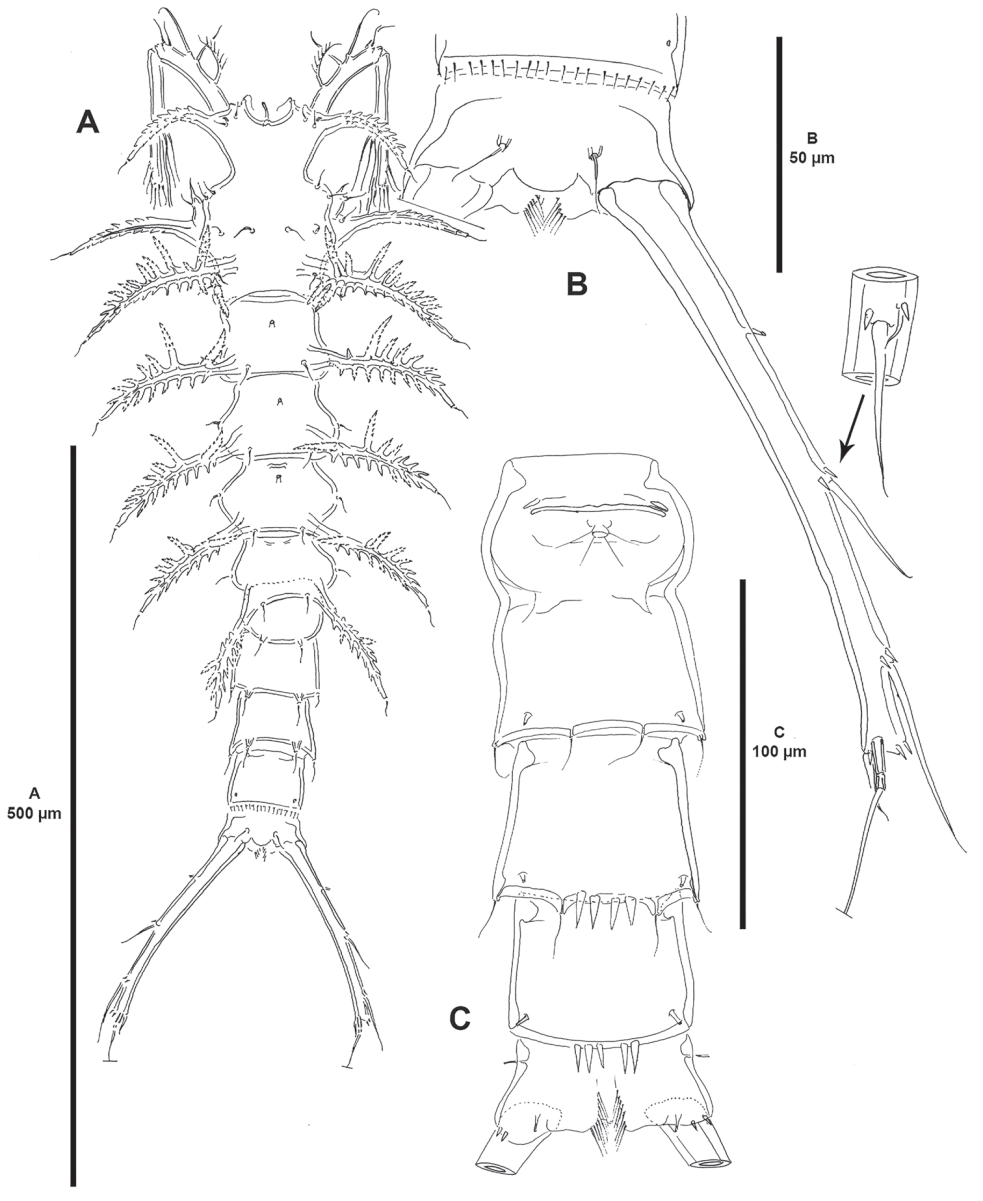
*Dendropsyllus californiensis* sp. n., female holotype. **A** habitus, dorsal **B** anal somite and right caudal ramus, dorsal, showing insert of lateral view of seta I and II **C** urosome, ventral, P5 bearing-somite and caudal rami omitted.

**Figure 8.**
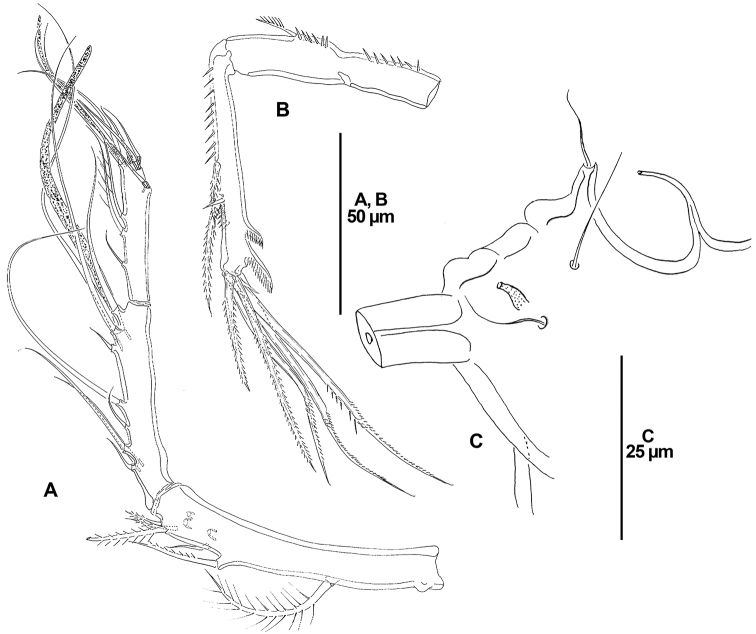
*Dendropsyllus californiensis* sp. n., female holotype. **A** antennule **B** antenna **C** distal outer corner of cephalothorax.

Total body length, 670 µm measured from anterior outer corner of cephalothorax to posterior margin of caudal rami; length of caudal rami, 145 µm (ca. 22% total body length). Body cylindrical, tapering posteriorly, without clear demarcation between prosome and urosome; integument moderately chitinised; well-developed dendroid processes as for the genus (Fig. 7A). Cephalothorax with bilateral anterior constriction (Fig. 7A); anterior corners with sensory triplet consisting of two sensilla and associated tube-pore (Fig. 8C); with paired sensillate processes as follows: paired dorsal dentate conical processes anteriorly, pair of dentate processes lateroventrally accompanied by anterior small sensillum-bearing processes, and paired dorsal dendroid processes posteriorly seemingly without tube-pore (Fig. 7A). Rostrum fused to cephalothorax, absorbed into anteroventral surface of cephalothorax, with paired sensilla-bearing tubercles, and with well-developed midventral tube-pore (Figs 7A, 8C). P2-P4-bearing somites with conspicuous dorsal tube-pore; with paired dorsal dendroid processes as shown, each with a sensillum halfway along length of process (Fig. 7A). P5-bearing somite seemingly without dorsal tube-pore; with paired dorsal dendroid processes less developed than in preceding somites.

Second and third urosomites fused ventrally forming genital double-somite, distinct dorsally, with dorsal sensilla-bearing tubercles as shown (Fig. 7A, C); proximal half (second urosomite) of genital double somite without sensory ornamentation, genital field as shown (Fig. 7C); distal half (third urosomite) of genital double-somite with paired tube-pores and posterior sensilla as shown. Fourth urosomite with dorsal and ventral sensilla as shown, with set of four strong ventral spinules medially (Fig. 7C). Fifth urosomite without sensilla, posterior margin with fine spinules (Fig. 7A, B), dorsally with paired pores as shown, ventrally with set of medial strong spinules close to posterior margin (Fig. 7C). Anal somite (Fig. 7A, B, C) partly cleft medially; dorsally with rounded and smooth anal operculum, and two sensilla; with two anterolateral, and two posteroventral tube-pores; with few small spinules on ventral hind margin.

Caudal rami (Fig. 7A, B) elongate, divergent, close to 7.5 times as long as wide; with lateral tube-pore on proximal third of ramus; ornamented with spinules as shown; with seven setae; seta I and II arising half way along lateral margin of ramus, the former minute and ventral to the latter; seta III somewhat longer than seta II, arising in distal seventh; setae IV and V broken off in Fig. 7B; seta VI small, arising on distal inner corner; dorsal seta VII triarticulate, situated close to distal margin of ramus.

Antennule (Fig. 8A) three-segmented, segments elongate and slender. Armature formula as follows: 1-[9], 2-[8+(1+ae)], 3-[8+acrothek].

Antenna (Fig. 8B), with allobasis; original division of basis and first endopodal segment indicated by membranous insert; basal and endopodal halves with small inner spinules as shown; endopodal half with one reduced abexopodal seta. Exopod absent. Free endopodal segment with inner spinules and two pinnate spines; outer margin with two frills subdistally; apically with two pinnate spines, two pinnate geniculate setae, and one pinnate geniculate seta with additional outer spinules halfway its length and fused basally to small seta.

**Figure 9.**
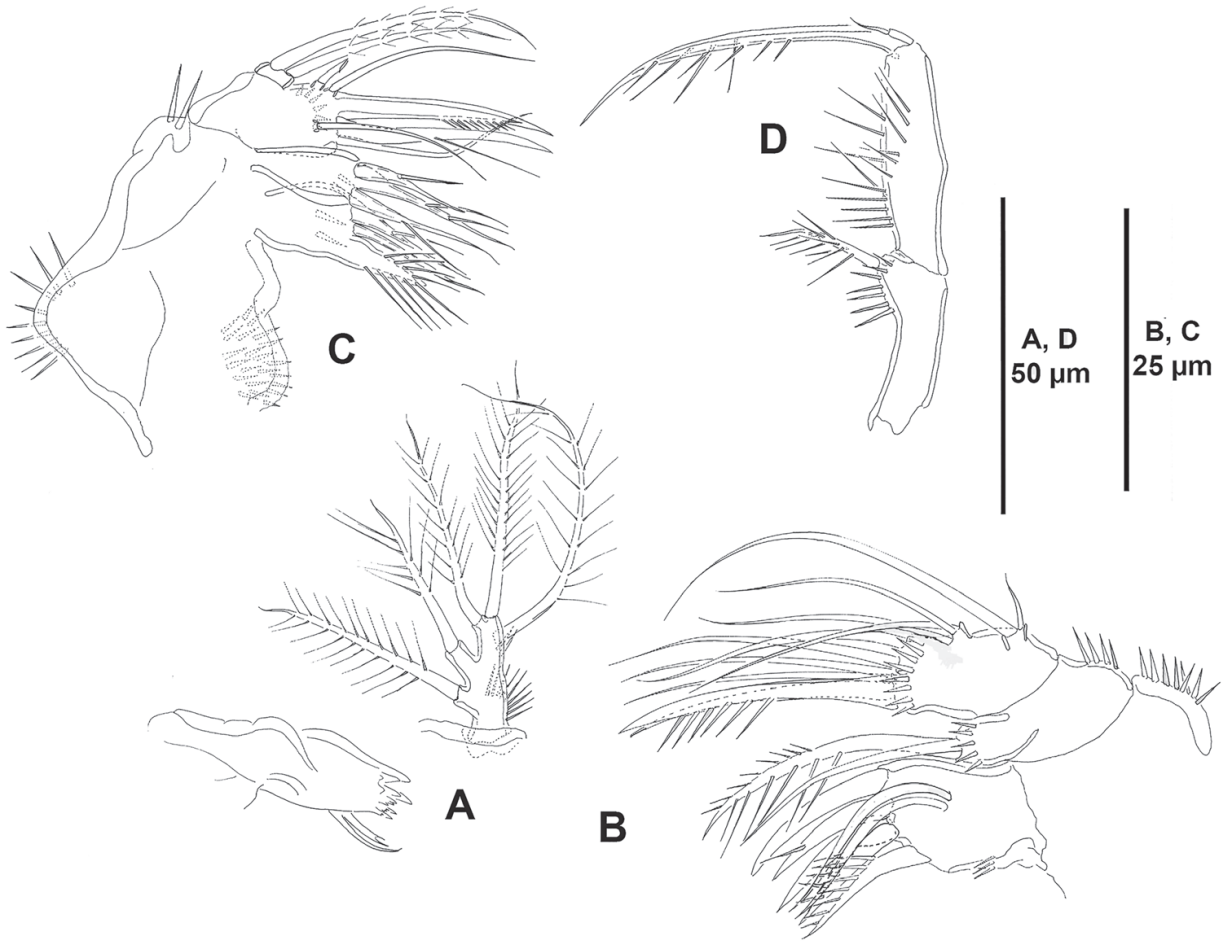
*Dendropsyllus californiensis* sp. n., female holotype. **A** mandible, showing detached palp **B** maxillule **C** maxilla **D** maxilliped.

Mandible (Fig. 9A) with robust coxa; gnathobase with teeth as figured, with two setae one of which bifid. Palp one-segmented, with spinules as shown, with two inner (basal), and three apical (endopodal) setae.

Maxillule (Fig. 9B) with quadrate praecoxal arthrite bearing two surface setae and five distal spines. Coxal endite with one spinulose and one bare seta, with some spinules distally. Proximal endite of basis with four, distal endite with two setae. Exopod represented by one long and one tiny seta. Endopod represented by three elements.

Maxilla (Fig. 9C). Syncoxa with spinulose patches as depicted; with two endites; proximal endite with three setae, one of which spinulose and basally fused to endite; distal endite with three spinulose elements. Allobasis drawn out into strong claw, the latter with subdistal spinules, accompanied by two outer elements, one strong spine, and two naked setae. Endopod very small, one-segmented, with two setae.

Maxilliped (Fig. 9D) subchelate. Syncoxa with some inner spinules apically and one spinulose seta on distal inner corner. Basis with spinules as depicted. Endopod drawn out into long spinulose spine with one accessory seta.

**Figure 10.**
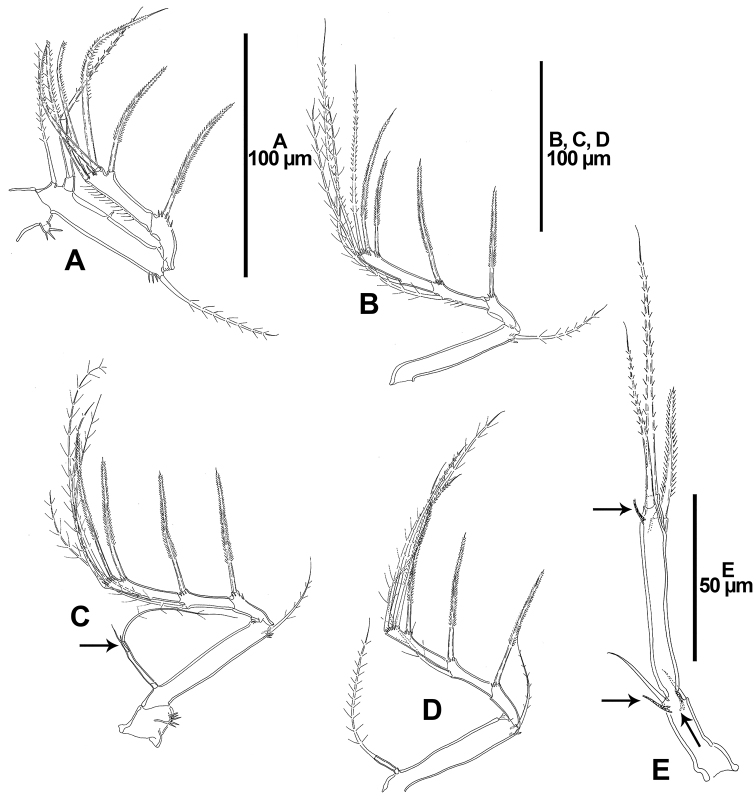
*Dendropsyllus californiensis* sp. n., female holotype. **A** P1, anterior **B** P2, anterior **C** P3, anterior, outer spine-like element of second endopodal segment indicated with an arrow **D** P4, anterior **E** P5, lateral, arrows showing tube pores.

P1 (Fig. 10A). Coxa trapezoid, with small lobate process bearing several spinules. Basis transversely elongate, with tube-pore midway along anterior margin, with one outer and one inner setae. Exopod two-segmented, ornamented with spinules and setules as depicted; first segment visibly shorter than second, with long outer pinnate spine; second segment elongate, without inner armature, with two apical geniculate setae, and with two outer geniculate elements and one bipinnate spine. Endopod two-segmented; first segment small, slightly longer than wide; second segment elongate, nearly 4.3 times as long as first segment, and almost 7.6 times as long as wide, with one apical seta.

P2-P4 (Fig. 10B–D). Coxa trapezoid, with outer lobate process ornamented with some spinules (as for P3, see Fig. 10C). Basis transversely elongate, with tube-pore close to outer seta, the latter bipinnate. Exopod three-segmented, exopodal segments with spinular ornamentation as shown; first segment without inner armature, with long bipinnate outer spine; second segment with inner seta and outer bipinnate spine; third segment of P2 and P3 with, of P4 without inner seta, with two apical setae and two outer bipinnate spines. Endopod of P2 absent; endopod of P3 and P4 two segmented, first segment very small, nearly as long as wide, second segment elongate, the latter 8.6 and 4.4 times as long as first segment and 8.6 and 4 times as long as wide in P3 and P4, respectively.

Armature formula as follows:

**Table:** 

	EXP	ENP
P1	0.023	0.010
P2	0.1.122	absent
P3	0.1.122	0.021
P4	0.1.022	0.010

P5 (Fig. 10E) with fused baseoendopod and exopod; outer basal seta naked, with accompanying tube-pore. Endopodal lobe represented by small pedestal with one naked seta accompanied by tube-pore. Exopod slender, elongate, 7.7 times as long as wide, with long subdistal tube-pore, with one outer, one distal (longest) and one inner (shortest) element.

######## Description of male.

Unknown.

######## Etymology.

The specific epithet, *californiensis*, makes reference to the Gulf of California, where the species was found.

## Discussion

Conroy-Dalton and Huys (2000) defined the *Ancorabolus*-group (*Ancorabolus*, *Arthropsyllus*, *Breviconia*, *Juxtaramia* and *Uptionyx*) and one year later, Conroy-Dalton (2001) defined the *Ceratonotus*-group composed by nine species and one subspecies within the genera *Ceratonotus*, *Dorsiceratus* and *Polyascophorus*, sharing a suite of seven synapomorphies (Conroy-Dalton 2001: 182). In that same paper, Conroy-Dalton (2001) provided enough evidence to support the monophyly of the genus *Ceratonotus* for which she identified nine apomorphies (Conroy-Dalton 2001, Fig. 8, Table 1) and divided the genus into two geographically separated clades, the sub-Antarctic *antarcticus-magellanicus* pair composed of *Ceratonotus antarcticus* George & Schminke, 1998 and *Ceratonotus magellanicus* George & Schminke, 1998 described from the Straits of Magellan (Chile) and an European group composed of *Ceratonotus coineaui* Soyer, 1965, *Ceratonotus pectinatus pectinatus* Sars, 1909, and *Ceratonotus pectinatus elaphus* Por, 1965 (Conroy-Dalton 2001: 187, Fig. 8). The *antarcticus-magellanicus* pair was defined by the relative size and general shape of the processes on the cephalic shield, the armature formula of the P1ENP (with one seta only), the loss of the P2ENP, and the lack of the inner seta on the P4EXP3 (and probably the P3EXP3 also) (Conroy-Dalton 2001). On the other hand, the European group was defined by the presence of dorsal dendroid processes on the posterior half of the female genital double-somite (Conroy-Dalton 2001).

Later, Conroy-Dalton (2003) created the genus *Dendropsyllus* based on the combination of seven apomorphies (Conroy-Dalton 2003: 92) to accommodate *Ceratonotus magellanicus* (= *Dendropsyllus magellanicus*) and *Ceratonotus antarcticus* (= *Dendropsyllus antarcticus*) from the Straits of Magellan, and a new species, *Dendropsyllus thomasi*, found near the base of the Coronado Escarpment, San Diego Trough in the north Pacific Ocean (Conroy-Dalton 2003). Also, Conroy-Dalton (2003) presented the diagnosis for the genus *Ceratonotus* in which she included the Norwegian type species, *Ceratonotus pectinatus*, *Ceratonotus coineaui* from Banyuls-sur-mer (French Mediterranean coast), and two new species, *Ceratonotus concavus* Conroy-Dalton, 2003 from the East Mediterranean coast of Hadera (Israel), and *Ceratonotus thistlei* from the Coronado Escarpment (San Diego Trough, north Pacific Ocean). Still later, George (2006a, b, c) published a series of articles on the taxonomy and systematics of the Ancorabolinae. In George (2006b), three new species of *Ceratonotus* are described, *Ceratonotus tauroides* George, 2006 from the Arctic Laptev Sea, and *Ceratonotus steiningeri* George, 2006 and *Ceratonotus vareschii* George, 2006 from the Angola deep-sea basin in the south Atlantic off Namibia, as well as the male of *Dendropsyllus magellanicus* found in the Chilean Pacific continental slope off Chiloé Island. As a result, George (2006b: 118) made some amendments to Conroy-Dalton’s (2003) generic diagnosis of *Ceratonotus* (at present composed of seven species, *Ceratonotus coineaui*, *Ceratonotus concavus*, *Ceratonotus steiningeri*, *Ceratonotus tauroides*, *Ceratonotus thistlei*, *Ceratonotus vareschii*, and its type species, *Ceratonotus pectinatus*), and reduced the number of apomorphies for the genus to only one, namely, the presence of dendroid dorsal processes on the male first abdominal somite (abdominal half of genital double-somite in the female). George (2006b) concluded also that the seven apomorphies identified for *Dendropsyllus* by Conroy-Dalton (2003) should be reduced to four, namely, the presence of four geniculate setae on P1EXP2, one seta only on P1ENP2, one inner seta only on P3EXP3, and lack of inner armature on P4EXP3. The other three apomorphies for *Dendropsyllus* identified by Conroy-Dalton (2003) turned out to be synapomorphies for *Ceratonotus* and *Dendropsyllus* (George 2006b: 120). Moreover, given the high number of synapomorphies for *Ceratonotus* and *Dendropsyllus*, and the low number of autapomorphies for each genus, George (2006b) questioned the preservation of the latter genus.

The first species proposed herein, *Ceratonotus elongatus* sp. n., can be undoubtedly attributed to *Ceratonotus* given a) the presence of dendroid processes on the posterior margin of the cephalothorax and on pedigerous somites 2-5, b) the presence of a simple conical lateroventral processes on each side of the cephalothorax, c) the presence of three geniculate setae on P1EXP2, d) the two-segmented condition of P2ENP, e) the presence of two apical setae on P2ENP2, f) the presence of two inner elements on P3EXP3, g) the discrete condition of the P5EXP, h) the insertion site of the caudal setae I and II (inserted in proximal third of caudal rami), and i) the presence of dendroid processes on the first abdominal somite (second half of the double genital-somite).

*Ceratonotus elongatus* sp. n. from the Guaymas Basin and *Ceratonotus thistlei* from the San Diego Trough are similar in several respects, and a close relationship between these two species is hypothesised. The description of *Ceratonotus elongatus* sp. n. is based on one female only, making the assessment of intraspecific variability impossible. Based on the present description, *Ceratonotus elongatus* sp. n. and *Ceratonotus thistlei* can be separated by the relative length of the two segments of the P2ENP (ENP2 1.6 times as long as ENP1 in *Ceratonotus thistlei*, but ENP2 nearly 2.7 times as long as ENP1 in the new species) and P4ENP (subequal in *Ceratonotus thistlei*, but ENP1 twice as long as ENP2 in *Ceratonotus elongatus* sp. n.), by the armature formula of the second antennulary segment (7+(1+ae) in the new species, but 6+(1+ae) in *Ceratonotus thistlei*), by the relative length of the caudal rami (11 times as long as wide in *Ceratonotus elongatus* sp. n., but nine times as long as wide in *Ceratonotus thistlei*), by the total body length (920 µm in *Ceratonotus elongatus* sp. n., but 677 µm in *Ceratonotus thistlei*), and above all, by the elongated sensilla-bearing dorsal tubercles on the second abdominal somite. Similar but somewhat smaller dorsal tubercles are known also for *Ceratonotus pectinatus*, and comparatively longer tubercles have been observed for *Ceratonotus concavus*, *Ceratonotus tauroides*, *Ceratonotus vareschii*, and *Ceratonotus steiningeri*, being extremely elongated in the latter. The presence of these tubercles is not exclusive for *Ceratonotus*, and similar but smaller tubercles have been observed also for *Dendropsyllus thomasi* and *Dendropsyllus magellanicus*. Similar tubercles have not been observed for *Ceratonotus coineaui*, *Ceratonotus thistlei*, and *Dendropsyllus antarcticus*. The dorsal dendroid processes of *Ceratonotus elongatus* sp. n. seems to be longer than in *Ceratonotus thistlei*. However, the comparatively shorter processes of *Ceratonotus thistlei* may be an artefact of the position of the body when observed in dorsal view, making the processes look shorter than they really are.

Conroy-Dalton (2003) did not observe any intraspecific variability in the four *Ceratonotus* females (one female holotype, and three female paratypes) upon which she based her description of *Ceratonotus thistlei*. Unfortunately, the description of *Ceratonotus elongatus* sp. n. is based on a single female and nothing can be said about the intraspecific variability of the species. Besides the differences noted above between *Ceratonotus thistlei* and *Ceratonotus elongatus* sp. n., the proposal of a new species of *Ceratonotus* from the Southern Trough of the Guaymas Basin is founded also on its relatively isolated situation, assuming that the Baja California Peninsula acts as an effective geographical barrier for the dispersal of these two species, thus preventing any gene flow.

*Ceratonotus elongatus* sp. n. and *Ceratonotus thistlei* can be separated from their congeners by a combination of several characters, i.e. the presence/absence of anterior horn-like processes of the cephalothorax (absent in *Ceratonotus elongatus* sp. n., *Ceratonotus thistlei*, *Ceratonotus coineaui* and *Ceratonotus pectinatus*, but present in *Ceratonotus tauroides*, *Ceratonotus steiningeri*, *Ceratonotus vareschii* and *Ceratonotus concavus*), the general shape and degree of development of the dorsal dendroid processes (visibly more developed in *Ceratonotus elongatus*, *Ceratonotus thistlei*, and *Ceratonotus concavus*, than in the other species), the armature formula of the second and third antennulary segments of the female (the females of *Ceratonotus concavus* and *Ceratonotus vareschii* remain unknown; the armature formula of second segment, 7+(1+ae) in *Ceratonotus elongatus* and *Ceratonotus tauroides*, but 6+(1+ae) in *Ceratonotus pectinatus*, *Ceratonotus thistlei*, and *Ceratonotus steiningeri*; the armature formula of third segment, 9+acrothek in *Ceratonotus elongatus*, *Ceratonotus pectinatus*, and *Ceratonotus thistlei*, but 8+acrothek in *Ceratonotus tauroides* and *Ceratonotus steiningeri*), by the spinulose nature of the outer margin of the endopodal segment of the antenna (without spinules in *Ceratonotus elongatus*, *Ceratonotus thistlei*, *Ceratonotus coineaui*, and *Ceratonotus pectinatus*, but with dense patch of fine spinules in *Ceratonotus tauroides*, *Ceratonotus steiningeri* and *Ceratonotus vareschii*), the number of elements on the first endite of the maxillary syncoxa (with two elements in *Ceratonotus vareschii*, but with three elements in the other species), by the nature of the spinular ornamentation of the basis of the maxilliped (densely covered with fine spinules in *Ceratonotus vareschii* and *Ceratonotus steiningeri*, but with comparatively fewer spinules in the other species), by the general shape of their antennules and antennary segments (comparatively more elongate and slenderer in *Ceratonotus elongatus* sp. n. and *Ceratonotus thistlei* than in the other species), the relative length of the outer basal element of P1 (visibly longer than basis in *Ceratonotus elongatus* sp. n., *Ceratonotus thistlei*, *Ceratonotus tauroides*, *Ceratonotus vareschii*, and probably *Ceratonotus steiningeri*, but relatively shorter in the other species), by the presence/absence of P2ENP (present in *Ceratonotus elongatus* sp. n., *Ceratonotus thistlei*, *Ceratonotus coineaui*, *Ceratonotus pectinatus*, *Ceratonotus concavus*, *Ceratonotus tauroides*, and *Ceratonotus vareschii*, but absent in *Ceratonotus steiningeri*), by the one- or two-segmented condition of P4ENP (one-segmented in *Ceratonotus coineaui* and *Ceratonotus pectinatus*, but two-segmented in *Ceratonotus elongatus* sp. n., *Ceratonotus thistlei*, *Ceratonotus tauroides*, *Ceratonotus steiningeri*, *Ceratonotus vareschii* and *Ceratonotus concavus*), by the armature formula of P4ENP (with one seta on P4ENP2 in *Ceratonotus elongatus* sp. n., *Ceratonotus thistlei*, *Ceratonotus tauroides*, and *Ceratonotus steiningeri*, with two setae on P4ENP2 in *Ceratonotus concavus* and *Ceratonotus vareschii*, and with one seta on the only segment of P4ENP in *Ceratonotus coineaui* and *Ceratonotus pectinatus*), and by the relative length of the caudal rami (11 times as long as wide in *Ceratonotus elongatus* sp. n. and *Ceratonotus steiningeri*, but nine and eight times as long as wide *Ceratonotus vareschii* and *Ceratonotus concavus*, respectively, 7.6 times as long as wide in *Ceratonotus thistlei*, and 6.8, 6.6 and 6.5 times as long as wide in *Ceratonotus coineaui*, *Ceratonotus pectinatus* and *Ceratonotus tauroides*, respectively).

The second species proposed herein, *Dendropsyllus californiensis* sp. n., has been unequivocally placed within the genus *Dendropsyllus* given a suite of characters defined by George (2006b: 120) as the only four apomorphies for the genus, namely, the presence of four geniculate setae on P1EXP2 and only one seta on P1ENP2, one inner seta only on P3EXP3, and lack of inner armature on P4EXP3. As noted above, the genus *Dendropsyllus* is composed of three species only, *Dendropsyllus antarcticus* from the Straits of Magellan (George and Schminke 1998), *Dendropsyllus magellanicus* known from the Straits of Magellan (George and Schminke 1998) and from the Chilean Pacific continental slope off Chiloé Island (George 2006b), and *Dendropsyllus thomasi* known only from the base of the Coronado Escarpment in the San Diego Trough (Conroy-Dalton 2003). Conroy-Dalton (2003) suggested a close relationship between *Dendropsyllus thomasi* and *Dendropsyllus magellanicus* based on the nature of the anterolateral and lateroventral cephalic processes, the spinulose nature of the maxilliped, the two-segmented P4ENP, and general shape of the female P5 (fused condition of the baseoendopod and exopod). Nevertheless, Conroy-Dalton (2003) was able to separate *Dendropsyllus thomasi* from the other two congeners by the elongate dendroid body processes (comparatively longer in *Dendropsyllus thomasi*, than in the Chilean species), by the slender and elongate first antennulary segment (7.8, 4.9, and 7.1 times as long as wide in *Dendropsyllus thomasi*, *Dendropsyllus magellanicus*, and *Dendropsyllus antarcticus*, respectively), and by the extreme elongation of the caudal rami (12.8, 8, and 7 times as long as wide in *Dendropsyllus thomasi*, *Dendropsyllus magellanicus*, and *Dendropsyllus antarcticus*, respectively). *Dendropsyllus californiensis* sp. n. seems to be more closely related to *Dendropsyllus thomasi* and *Dendropsyllus magellanicus* than to *Dendropsyllus antarcticus* on account of the spinulose nature of the basis of the maxilliped, the two-segmented P4ENP, and the fused condition of the P5 baseoendopod and exopod. On the other hand, *Dendropsyllus californiensis* sp. n. seems to be more related to *Dendropsyllus thomasi* by the degree of development of the lateroventral processes of the cephalothorax which seem to be longer than the posterodorsal processes in these two species than in the two Chilean representatives. The new species proposed herein, *Dendropsyllus californiensis* sp. n., can be separated from its congeners by the relative length of the first segment of the antennule (6.5 times as long as wide) and by the relative length of the caudal rami (7.5 times as long as wide), and above all, by the presence of a small outer spine-like element on the second endopodal segment of P3. Note that in Fig. 10C, this element appears to be located just in front of the segment. This could be an artefact of the mounting process since this spine-like element is clearly situated along the outer margin of the segment as observed on the not dissected P3.

The genus *Dendropsyllus*, when found, occurs at very low densities (for example, one single specimen of *Dendropsyllus californiensis* sp. n. was found in the present study), and most species of the genus are known from one sex only (Conroy-Dalton 2003), which prevents any phylogenetic analysis (George 2006b). So far, *Dendropsyllus magellanicus* is the only species for which both sexes are known. The species was originally described based on one female only (George and Schminke 1998), and some years later George (2006b) described the male. With this record, George (2006b) was able to observe the expression of sexual dimorphism in the antennule (six-segmented, subchirocer), P3ENP (three-segmented, with long inner apophysis on ENP2), P4ENP (two-segmented, both segments small and subequal), and P5 (baseoendopod and exopod separated). The outer spine-like element observed on the P3ENP2 of *Dendropsyllus californiensis* sp. n., is considered here as a novel, autapomorphic element for the species and not homologous to the inner apophysis observed for the male of *Dendropsyllus magellanicus*.

The formation of the Gulf of California is a very recent and complicated process that began between 130 and 90 mya during the Cretaceous when the Farallon Plate started to subduct eastward from the East Pacific Rise under the North American Plate, while the latter was moving slowly westward (Ledesma-Vázquez and Carreño 2012). Following Ledesma-Vázquez and Carreño’s (2012) scheme, it seems reasonable to hypothesise that during the Cretaceous, the location where Conroy-Dalton (2003) found *Ceratonotus thistlei* and *Dendropsyllus thomasi* was situated 330–400 km south of its current position. This suggests that these two species may be present off the entire Baja California Peninsula. The original populations of these species may have invaded the northern Gulf of California through an early proto-Gulf of California marine incursion during the early late Miocene from 14 to 12 mya (Helenes and Carreño 1999, Ledesma-Vázquez 2002) and during the first great incursion of the Pacific Ocean in the late Miocene-earliest Pliocene 8.2-7.5 mya (Ledesma-Vázquez and Carreño 2012). Following Nagy and Stock (2000), spreading of the Gulf of California began in the early Pliocene around the mouth of the Gulf, and the Guaymas Basin opened 2.1 mya. If this scenario is assumed to be correct, the speciation of the genera *Ceratonotus* and *Dendropsyllus* may have been potentiated on one hand, by the formation of the Gulf of California and subsequent geographic isolation by the consolidation of the Baja California Peninsula, and on the other hand, by the subsequent movement of the Pacific Plate north-westwards.

## Supplementary Material

XML Treatment for Ceratonotus elongatus

XML Treatment for Dendropsyllus californiensis
